# Views of Emergency Physicians on Thrombolysis for Acute Ischemic Stroke

**DOI:** 10.4137/jcnsd.s2231

**Published:** 2009-03-30

**Authors:** Bentley J. Bobrow, Bart M. Demaerschalk, Joseph P. Wood, Albert Villarin, Lani Clark, Anthony Jennings

**Affiliations:** 1 Department of Emergency Medicine Mayo Clinic, Scottsdale, Arizona.; 2 Department of Neurology, Mayo Clinic, Scottsdale, Arizona.; 3 Albert Einstein Medical Center, Philadelphia, Pennsylvania.; 4 Arizona Department of Health Services, Bureau of Emergency Medical Services, Phoenix, Arizona.; 5 St. Joseph’s Hospital, Lake St. Louis, Missouri.

**Keywords:** acute ischemic stroke, emergency physician, primary stroke center, thrombolysis, stroke neurologist

## Abstract

**Background:**

The 3-hour window for treating stroke with intravenous tissue plasminogen activator (t-PA) requires well-organized, integrated efforts by emergency physicians and stroke neurologists.

**Objective:**

To evaluate attitudes and knowledge of emergency physicians about intravenous t-PA for acute ischemic stroke, particularly in primary stroke centers (PSCs) with stroke neurology teams.

**Methods:**

A 15-question pilot Internet survey administered by the Arizona College of Emergency Physicians.

**Results:**

Between March and August 2005, 100 emergency physicians responded: 71 in Arizona and 29 in Missouri. Forty-eight percent practiced at PSCs; 48% thought t-PA was effective, 20% did not, and 32% were uncertain. PSC or non-PSC location of practice did not influence endorsement (odds ratio, 0.96; 95% confidence interval, 0.27–1.64). Of those opposing t-PA, 87% cited risk of hemorrhage.

**Conclusions:**

Most emergency physicians did not endorse t-PA. Improved collaboration between emergency physicians and stroke neurologists is needed.

## Introduction

In 1995, the National Institute of Neurological Disorders and Stroke (NINDS) Stroke Study Group reported that intravenous recombinant tissue plasminogen activator (t-PA) produced an 11% to 13% absolute and a 30% to 50% relative improvement in complete or almost complete neurologic recovery in patients with acute ischemic stroke, albeit with a 6.4% risk of symptomatic intracerebral hemorrhage.^1^ The following year, the U.S. Food and Drug Administration approved the use of intravenous t-PA for treatment of patients with ischemic stroke within the first 3 hours of symptom onset.^1^

Despite the availability of t-PA since 1996, still only a low proportion (1%–2%) of acute ischemic stroke patients nationwide receives it.^2^ Several reasons for this lack of use exist, including a failure on the part of the general public to recognize the signs and symptoms of stroke, to understand that stroke is a time-sensitive emergency, and to immediately access emergency medical services by calling 9-1-1.^3^ Cumulatively, these factors often lead to the delayed presentation of the acute stroke patient to an emergency department and thus to limited treatment options.^3^ Furthermore, the lack of a uniform position among emergency physicians on the role of acute thrombolysis with intravenous t-PA adds to the low rate of thrombolysis in eligible patients with acute ischemic stroke.^4^–^6^

A recent national Internet survey on thrombolysis for acute ischemic stroke showed that physicians in emergency departments were reluctant to use t-PA.^7^ As many as 40% would not administer intravenous recombinant rt-PA for acute ischemic stroke, even under ideal conditions, largely because of the risk of symptomatic intracerebral hemorrhage.

We sought to collect pilot data which would help begin to identify and prioritize the specific concerns of emergency physicians about t-PA treatment of acute ischemic stroke. We also sought to discern whether their concerns were affected by the immediate availability of acute stroke neurology teams and the implementation of a formal designation of area hospitals and medical centers as primary stroke centers (PSCs). In addition, we sought to elucidate the knowledge about, and satisfaction with, the existing local matrix of 8 PSCs among emergency department physicians within the Phoenix Metropolitan Matrix of PSCs.

## Methods

This study used an anonymous, voluntary, 15-question Internet survey administered by the Arizona chapter of the American College of Emergency Physicians (ACEP) on its official Web site between March 1 and August 31, 2005. The survey gathered demographic information and opinions about the use of intravenous t-PA for acute ischemic stroke. Each of the approximately 430 members of Arizona ACEP along with the approximately 350 members of Missouri ACEP were invited by e-mail to participate in the Internet survey. Missouri ACEP leadership was interested in participating in this Internet survey along with Arizona ACEP who were the primary survey target. We used this as an opportunity to collect data on possible regional variations of respondents. Survey items were validated using health measurement survey methodology.^8^ The background literature was searched, many potential survey items were devised, scaling responses were developed, final survey items were selected based on pre-testing with a small sample of emergency physicians, and the preferred method of survey administration was chosen. ACEP members in Arizona and Missouri regularly receive communications via this internet site. This study was approved by the Human Subject Research Board of the Arizona Department of Health Services.

### Selection of participants

During a period of 3 months, 3 separate e-mail messages were sent to members of the Arizona chapter of ACEP to direct them to the Internet survey located on the chapter’s Web site. A printed copy of the 15-question survey was also included in the monthly newsletter of the Arizona chapter of ACEP to encourage members to take the survey either on the chapter’s Web site or by completing the paper copy of the survey and sending it in by fax.

During the same 3-month period, the Missouri College of Emergency Physicians also posted the URL (uniform resource locator) link to the acute stroke survey on its chapter Web site.

A copy of the 15-question Internet survey with potential answer selections is attached as an Appendix. No additional definitions or directions were provided.

### Outcome measurements

The primary outcome measurements were: 1) The proportion of emergency physicians who were practicing in conjunction with stroke neurology teams; 2) The proportion of emergency physicians who endorsed the use of intravenous t-PA for acute ischemic stroke under ideal conditions; 3) The association between practicing in conjunction with stroke neurology teams and endorsement of t-PA; 4) The proportion of emergency physicians willing to administer intravenous t-PA under the guidance of a stroke neurologist by telemedicine; and 5) The baseline awareness of emergency physicians in Arizona about the existing Phoenix Metropolitan Matrix of PSCs.

### Primary data analysis

Demographic responses were tabulated, and the proportions of respondents who endorsed specific responses were determined. An odds ratio with 95% confidence interval was calculated to reflect the association between PSC location of practice and endorsement of t-PA.

## Results

### Characteristics of study participants

A total of 100 emergency physicians completed the survey between March and August 2005. Of all 100 respondents, most (92%) indicated that they were full-time emergency physicians.

Most (71%) of the survey respondents stated that they were currently practicing emergency medicine in Arizona. The rest (29%) practiced in Missouri.

Most (68%) of the survey respondents indicated that they had residency training in emergency medicine. Slightly more (77%) were allopathic physicians by training, and (23%) had received osteopathic medical training.

More than one-third of the respondents (39%) reported that they had been practicing medicine for longer than 15 years. In contrast, only slightly more than one-fourth (26%) had been practicing for fewer than 5 years. The rest of the survey respondents had been in practice 5 to 10 years (14%) or 11 to 15 years (20%).

Slightly less than half (48%) of the 100 emergency physicians who responded to the survey reported that they practiced at a hospital designated as a PSC.

Forty-eight of the emergency physicians who completed the survey reported that they viewed administration of intravenous t-PA per the NINDS protocol as an effective treatment for acute ischemic stroke. A comparable number (51%) endorsed the use of t-PA for eligible patients with acute ischemic stroke. In contrast to the 48% who believed t-PA to be effective, however, 32% of the respondents indicated that they were uncertain about its effectiveness and 20% said that they did not believe it was effective. Similarly, 32% said that they did not endorse its use and 16% were undecided about doing so. There was no statistically significant difference in endorsement of intravenous recombinant t-PA among those emergency physicians in PSCs versus non-PSCs (odds ratio, 0.96; 95% confidence interval, 0.27–1.64).

If respondents answered “no” or “uncertain” to either question 7 or question 8, they were asked to indicate their reasons for not endorsing t-PA for patients with acute ischemic stroke. Of the 47 emergency physicians who did not endorse intravenous t-PA, 87% gave their reasons as risk of hemorrhagic complications and 70% as lack of proven efficacy (Table 1). However, about half the respondents who did not endorse t-PA indicated that they were hesitant to do so because of a lack of neurology (51%) or radiology (45%) support.

Of the 71 Arizona emergency physicians who completed the survey, only 34% were aware of the existence of the Phoenix Metropolitan Matrix of PSCs (Table 2). Sixty-five percent were not aware of this coalition.

The respondents who indicated that they were not aware of the Phoenix Metropolitan Matrix of PSCs were asked whether they had a similar system of PSCs in their own city. The most common response for 49% of the respondents was uncertainty as to whether there was a similar system in their city, and 28% said that there was not; only 17% reported having access to a similar system.

Only 49% of the Arizona respondents indicated that they were aware of the EMS practice of triaging suspected acute stroke patients to a PSC. Forty-five percent of respondents were not aware of this practice, and 6% were uncertain that they had heard of it.

Forty percent of the responding emergency physicians reported that they had stroke neurology teams at their hospitals (Table 3). Of those respondents with a stroke neurology team, 73% were either satisfied or very satisfied with the neurology service.

Sixty-two percent of participants indicated that they would be willing to administer intravenous t-PA to acute ischemic stroke patients after a formal consultation with a stroke neurologist by telemedicine. However, 17% would still not be willing to do so and 20% were still uncertain about the advisability of this course of action.

In response to the final survey question, 74% of the emergency physicians said that they had treated more than 5 acute stroke patients in the emergency department during the past 12 months. Only 4% reported not treating any stroke patients.

### Limitations

There are several limitation to this study, with the primary one being the lack of participation. Despite recontacting physicians we were only able to sample a small proportion of EPs in this pilot survey. Only 71 of approximately 430 (16.5%) emergency physicians in Arizona responded to either the Internet (n = 69) or printed version (n = 2) of the survey. To maximize response rates, we kept the survey simple and brief. The brevity of the survey questions may have caused some ambiguity for the participants. For example, some respondants may only view IV tPA as efficacious if its administration improves survival as opposed to if morbidity is improved with its use. We also did not differentiate between emergency medicine residents and attending physicians but since most ACEP members have completed training and are in practice, we believe that the number of emergency medicine residents responding to the survey was very low.

Because only 29 emergency physicians from the Missouri Chapter of Emergency Physicians participated, it is difficult to draw meaningful conclusions from this subset of study participants. Nonetheless, their responses added depth to the overall findings of the survey.

## Discussion

Ischemic stroke is a devastating illness of enormous proportions in the United States, where it is the leading cause of disability.^9^–^11^ Although several treatment options are on the horizon, the U.S. Food and Drug Administration has currently approved only one medication (intravenous t-PA) for acute treatment of ischemic stroke. The average use of that treatment is just 1% to 2%.^2^ Myriad reasons account for this low rate of use. Although administration of t-PA does not equate to overall stroke care, it may be used as a surrogate marker. The findings with this pilot survey were consistent with the low rates of intravenous thrombolysis still found in most areas of the country.

Emergency physicians are essential providers in the chain of acute medical care required for optimal treatment of patients with acute ischemic stroke. The brief 3-hour window of eligibility for treatment with intravenous t-PA requires a well-organized and well-integrated effort by emergency physicians and stroke neurologists.

In 1998, the American Stroke Association Phoenix Operation Stroke partnered with the Arizona Emergency Medical Systems in a county-wide collaboration with the goal of increasing to the proportion of acute stroke patients receiving tissue plasminogen activator.

A system was established for metropolitan prehospital emergency medical providers to identify, give prenotification and transport patients with acute stroke to a predesignated PSCs. During the ensuing eight years, emergency medical system personnel were trained to identify and transport patients with acute stroke to one of eight PSCs in the metropolitan Phoenix Area. The system became operational (in 2003), and 18% of patients with acute ischemic stroke received thrombolysis, thus demonstrating that it is feasible to develop and operationalize a successful metropolitan-wide matrix of PSCs to accommodate a 9000-square-mile region with a population of 3.5 million people.^12^

No uniform template exists for how emergency physicians and stroke neurologists should best collaborate to improve the number of eligible patients receiving intravenous t-PA. Although some PSCs have stroke neurology teams able to consistently respond in person to their emergency department, this approach is not universal. Consequently, the neurologist who is on call for acute stroke may not be immediately available for consultation. In fact, Brown et al^13^ documented that only 8% of patients with acute ischemic stroke or transient ischemic attack received an in-person neurology consultation in a community-based emergency department. Yet, given that stroke is a neurologic emergency, it is unreasonable to expect emergency physicians to shoulder the burden of care for direct neurology support of acute stroke patients. Our findings indicated that more than half of the emergency physicians cited a lack of neurology support as a reason for not endorsing the use of intravenous t-PA for acute ischemic stroke. How best to structure the relationship between emergency physicians and neurologist has yet to be determined.

Unfortunately, emergency physicians in community hospital settings are often the on-site caregivers solely responsible for deciding whether to administer thrombolytic therapy. Emergency physicians have been found to be quite accurate in their ability to diagnose stroke.^14^ However, depending on their practice location, many of these physicians may face clinical situations only a few times each year when intravenous t-PA might be indicated.^14^

No doubt the lack of uniform support by national emergency medicine organizations contributes to the low usage rates for intravenous t-PA across the United States. Although ACEP states that this form of thrombolytic therapy may be beneficial when adequate institutional support is in place, neither the American Academy of Emergency Medicine^5^ nor the Society for Academic Emergency Medicine^6^ supports the routine use of intravenous t-PA for acute ischemic stroke as the standard of care. Indeed, our survey results confirm that such reluctance is the status quo, with only 52% of all respondents endorsing intravenous t-PA for eligible acute ischemic stroke patients even under ideal conditions. Nonetheless, we would dare to suggest that emergency medicine organizations have both an opportunity and an obligation to participate in defining the standard collaboration model.

More and more, the success of t-PA as a clot-busting medication is becoming better known among the general public. Experience at our institution is that more patients are now demanding the option of acute treatment for ischemic stroke than in the past. This increased consumer demand often leaves practicing emergency physicians in the difficult position of arbitrating between what their professional organizations recommend and what their patients expect.

Our results indicate that just 48% of the emergency physicians who responded to the survey view t-PA as effective and that only 52% would endorse its use under ideal conditions. Although we cannot generalize these findings to the specialty as a whole, emergency physicians appear to hold the same opinion about the use of thrombolysis regardless of whether or not they work in a PSC.

The finding that 62% of emergency physicians might be willing to administer intravenous t-PA after telephone or teleconference consultation with a stroke neurologist is of keen interest. Thus, the delivery of appropriate medical care to acute stroke patients in rural settings should benefit from the continued development of, and increased reliance on, telemedicine.

Although emergency physicians are not uniformly supportive of the use of intravenous t-PA or PSCs, the development of the PSC continues to generate greater awareness and gain more acceptance. The Joint Commission on Accreditation of Healthcare Organizations (JCAHO) has certified almost 220 PSCs already since it first began surveying hospital stroke care programs in 2003.^15^ Additionally, other states have implemented the PSC designation through their public health departments. However, JCAHO certification and state designation of prospective PSCs are unlikely to result in an immediate increase in the proportion of stroke patients receiving acute stroke therapy. The ultimate goal of increasing delivery of acute stroke interventions will be accomplished only through a comprehensive practice model that standardizes the expectations of all the medical specialties involved in the acute care of these patients.

The evolution of the PSC model is still relatively new. Although the PSC concept, analogous to that of the trauma center, is intuitively sound, it has yet to be formally validated by documented improvements in patient outcomes.^16^ The growing number of PSCs in cities and communities nationwide has the potential to influence emergency department practices, help alleviate emergency department overcrowding, and affect the use of resources both within the emergency department and among emergency medical service systems. Emergency physicians must therefore strive to be continually informed and updated about the status of the PSCs developing within their communities.

In the past decade, numerous advances have been made in stroke prevention, treatment, and rehabilitation. 17,18 In addition to the currently approved acute stroke therapy of intravenous t-PA, other potential therapies include intra-arterial thrombolysis, mechanical clot removal (Mechanical Embolus Removal in Cerebral Ischemia [MERCI] Retriever; Concentric Medical, Inc, Mountain View, California), and other clot-busting and neuroprotective medications under development. As the various options for acute treatment expand, so will the practice of accurately identifying, stabilizing, and transporting acute stroke patients to PSCs. The keystone to substantial improvement in outcomes, however, will continue to be the partnership between emergency physicians and stroke neurologists.

## Conclusion

Regardless of affiliation with a PSC or a non-PSC, a significant proportion of most emergency physicians in this brief Internet survey did not endorse administration of intravenous t-PA for eligible patients with acute ischemic stroke. For optimal delivery of acute stroke care that includes this treatment option, it is imperative to establish a clear, effective, and realistic collaboration model for use by emergency physicians and stroke neurologists. Ongoing research into how regional variations and subsets of emergency physician populations may view this topic are needed.

## Figures and Tables

**Table 1:** Reasons for Not Using Intravenous Tissue Plasminogen Activator.*†

Reason	%
Risk of hemorrhagic complications	87
Lack of proven efficacy	70
Lack of neurology support	51
Other	47
Lack of radiology support	45
Drug cost	13

* Question 9: If you answered “no” or “uncertain” to either question 7 or 8, what are your reasons for not endorsing tissue plasminogen activator for acute ischemic stroke?

† N = 47 emergency physicians.

**Table 2:** Awareness of and satisfaction with phoenix metropolitan matrix of primary stroke centers.*†

Variable	%
Awareness	
Yes	34
No	65
Uncertain	1
Satisfaction	
Very satisfied	8
Satisfied	38
Neutral or uncertain	38
Dissatisfied	4
Very dissatisfied	12

* Question 10: Are you aware of the Phoenix Metropolitan Matrix of primary stroke centers? (Results from Arizona respondents only).

† N = 71 emergency physicians.

**Table 3:** Awareness of and satisfaction with hospital acute stroke neurology team.*†

Variable	%
Awareness	
Yes	41
No	53
Uncertain	6
Satisfaction	
Very satisfied	45
Satisfied	28
Neutral or uncertain	12
Dissatisfied	8
Very dissatisfied	2

* Question 13: Do you have an acute stroke neurology team at your hospital?

† N = 100 (71 from Arizona) emergency physicians.

## Abbreviations

ACEP: American College of Emergency Physicians
JCAHO: joint commission on accreditation of healthcare organizations
NINDS: national institute of neurological disorders and stroke
PSC: primary stroke center
t-PA: tissue plasminogen activator
URL: uniform resource locator.

## Appendix

**Table:**

Emergency Medicine Physician Acute Stroke Questionnaire*	
1) Are you a full-time emergency physician?	
Yes = 92	**92%**
No = 6	**6%**
NR = 2	**2%**
2) State in which you practice:	
Arizona = 71	**71%**
Missouri = 28	**28%**
NR = 1	**1%**
3) Are you residency trained in emergency medicine?	
Yes = 68	**68%**
No = 30	**30%**
NR = 2	**2%**
4) Are you an:	
MD = 77	**77%**
DO = 23	**23%**
5) Exclusive of residency training, how many years have you been practicing emergency medicine?	
Less than 5 = 26	**26%**
5–10 = 14	**14%**
10–15 = 20	**20%**
More than 15 = 39	**39%**
NR = 1	**1%**
6) Do you practice predominantly at a hospital that is a Primary Stroke Center (PSC)?	
Yes = 48	**48%**
No = 52	**52%**
7) Do you view intravenous t-PA administered per NINDS protocol as an effective treatment for acute ischemic stroke?	
Yes = 48	**48%**
No = 20	**20%**
Uncertain = 32	**32%**
8) In general, do you endorse t-PA for eligible acute ischemic stroke patients?	
Yes = 51	**51%**
No = 31	**31%**
Uncertain = 16	**16%**
NR = 2	**2%**
9) If you answered NO or UNCERTAIN to either question #7 or #8, what are your reasons for NOT endorsing t-PA for acute ischemic stroke?†	
Risk of hemorrhagic complications = 41	**87.2%**
Lack of proven efficacy = 33	**70.2%**
Drug cost = 6	**12.8%**
Lack of neurology support = 24	**51.1%**
Lack of radiology support = 21	**44.7%**
Other = 22	**46.8%**
10) Are you aware of the Phoenix Metropolitan Stroke Matrix?	
Yes = 28	**28%**
No = 71	**71%**
Uncertain = 1	**1%**
If YES, rate your level of satisfaction. (*N* = 28)	
Very satisfied = 3	**10.7%**
Satisfied = 12	**42.9%**
Neutral/uncertain = 9	**32.1%**
Dissatisfied = 1	**3.6%**
Very dissatisfied = 3	**10.7%**
11) If you are not aware of the PMSM, do you have a similar system of PSCs in your city? (*N* = 72)	
Yes = 12	**16.7%**
No = 20	**27.8%**
Uncertain = 35	**48.6%**
Aware of PMSM = 10	**13.9%**
NR = 5	**6.9%**
12) Are you aware that in the Phoenix Metropolitan Area, EMS personnel transport acute stroke patients that meet Operation Stroke criteria preferentially to PSCs?	
Yes = 41	**41%**
No = 55	**55%**
No response = 4	**4%**
13) Do you have an acute stroke neurology team at your hospital?	
Yes = 40	**40%**
No = 51	**51%**
Uncertain = 6	**6%**
NR = 3	**3%**
If YES, rate your level of satisfaction with the hospital’s acute stroke neurology team.	
Very satisfied = 18	**45%**
Satisfied = 11	**27.5%**
Neutral/uncertain = 5	**12.5%**
Dissatisfied = 3	**7.5%**
Very dissatisfied = 1	**2.5%**
14) Would you be willing to administer t-PA in the ED to acute stroke patients after formal consultation with a stroke neurologist via telemedicine?	
Yes = 62	**62%**
No = 17	**17%**
Uncertain = 20	**20%**
NR = 1	**1%**
15) How many acute stroke patients have you cared for in your ED in the past 12 months?	
None = 4	
1 = 2	
2 = 9	
3 = 5	
4 = 2	
5 = 4	
More than 5 = 74	
16) If practicing primarily at a PSC, do you endorse t-PA? (*N* = 48)‡	
Yes = 25	**52.1%**
No = 13	**27.1%**
Uncertain = 8	**16.7%**
NR = 2	**4.2%**
17) If practicing primarily at a non-PSC, do you endorse t-PA? (*N* = 52)	
Yes = 25	**48.1%**
No = 17	**32.7%**
Uncertain = 8	**15.4%**
NR = 2	**3.8%**

ED, emergency department; EMS, emergency medical service; NINDS, national institute of neurological disorders and stroke; NR, no response; t-PA, tissue plasminogen activator.

* N = 100 unless otherwise indicated.

† N = 99, but some respondents gave more than one answer.

‡ Total percentage equals more than 100 due to rounding.

Questions 1–15 are from the Arizona College of Emergency Physicians. The Emergency Medicine Physician Acute Stroke Questionnaire. c2005. Available from: http://www.azcep.org/azcep/stroke.htm. Used with permission.
